# The Use of Calcium Sulphate as a Drug Delivery System in Lower Limb Amputation

**DOI:** 10.7759/cureus.97839

**Published:** 2025-11-26

**Authors:** Geraint Morris, Raviprasad Kattimani, Noel Fisher

**Affiliations:** 1 Trauma and Orthopaedics, North Manchester General Hospital, Manchester, GBR; 2 Trauma and Orthopaedics, East Cheshire NHS Trust, Macclesfield, GBR

**Keywords:** amputation, antibiotic, calcium sulfate, calcium sulphate, osteomyelitis, revision

## Abstract

Background

Amputation in the lower limb can be a challenging procedure both for the surgeon and the patient. Common complications include revision of amputation and wound infection, increasing morbidity to patients and putting further pressure on stretched health services. We describe a simple technique used in lower limb amputation using an antibiotic-loaded calcium sulphate bone graft placed in the medullary canal and soft tissues to control haemostasis and deliver a high dose of antibiotics locally into the wound bed.

Methodology

We describe a series of 17 lower limb amputations in 15 patients from our unit between 2014 and 2021. Infection was eradicated through aggressive debridement of the infected bone and soft tissues. An antibiotic-loaded calcium sulphate bone graft was placed into the medullary canal, along with pellets into the soft tissues. Wounds were either closed primarily or left open to heal by secondary intention due to skin loss following debridement. Our primary outcome measure was the need for amputation revision to a more proximal level during the acute treatment phase (six weeks).

Results

Indication for amputation in most cases was osteomyelitis. Operations included below-knee, transmetatarsal, and ray amputations. In this 17-procedure series, no patients required revision of amputation to a more proximal level in the acute treatment phase using this technique. Overall, there were three incidences of wound infection (17.7%).

Conclusions

Placing calcium sulphate beads loaded with antibiotics into the medullary canal and surrounding tissues is a simple adjunct in lower limb amputation, aiming to reduce rates of wound infection and amputation revision. Comparing this cohort to previous literature is challenging due to mixed indications and levels of amputation. However, the outcomes described aid the pragmatic decision-making in a complex patient group.

## Introduction

Globally, lower limb amputation has an incidence of 16.9-22.9 per 10,000 [1]. After trauma, other common indications for amputation are diabetic foot, peripheral arterial disease, bone and joint infections, peripheral neuropathy, and malignancy [2,3].

Lower limb amputation is a life-changing procedure associated with considerable morbidity and mortality [3,4]. The most common complication has been reported to be wound infection, with others including phantom limb pain, stump problems (including bone prominence and ulceration), and venous thromboembolism [5]. Wound healing can be particularly problematic in diabetic patients. Following initial amputation, some patients require re-amputation; diabetic patients, in particular, had rates being reported as high as 26% at one year following lower limb amputation, rising further at two years to 45.9% [4].

Calcium sulphate beads have been used in the local delivery of antibiotics in chronic osteomyelitis, prosthetic joint infections, arthroplasty, open fractures, and combat injuries. They are absorbed following implantation, and the local antibiotic concentration is sustained over several weeks [6]. In amputation, studies have reported reduced wound breakdown when placed into the soft tissues during transmetatarsal amputation [7] and reduced recurrence of infection in forefoot amputations [8]. Calcium sulphate also acts as a bone graft substitute, stimulating osteogenesis, as it is both osteoconductive and osteoinductive [9]. In below-knee amputation, gentamicin-loaded calcium sulphate and hydroxyapatite have been used as a medullary canal plug, occluding the canal and preventing excessive bleeding alongside its antibiotic eluting qualities [10].

We describe a technique using calcium sulphate bone graft (Stimulan®) loaded with vancomycin and gentamicin placed in the medullary canal and the soft tissue of both minor amputations (below the level of the ankle) and major lower limb amputations (above the level of the ankle) to control haemostasis and deliver a high dose of antibiotics locally in the hope of reducing postoperative infection and time to heal.

This article was previously presented as a poster at the 2023 BIOS 24th Annual Conference on 7th-8th July 2023.

## Materials and methods

In this case series, we retrospectively reviewed clinical notes and electronic patient records of patients who underwent lower limb amputation under the care of one consultant orthopaedic surgeon in our unit between 2014 and 2021. Inclusion criteria were above-knee (transfemoral), below-knee (transtibial), midfoot (transmetatarsal), and ray amputations (partial transmetatarsal). Toe amputations and disarticulations were excluded.

All patients were operated under general anaesthesia, and intravenous broad-spectrum antibiotics were given at the time of induction. A tourniquet was used in all patients. Amputation was performed after aggressive debridement of the infected bone and soft tissue, followed by copious irrigation with saline to eradicate infection. Vancomycin 1 g and gentamicin 240 mg (five patients received solely vancomycin) were mixed with 5-10 cc of calcium sulphate bone graft (Stimulan®, Biocomposites Ltd, Staffordshire, UK), which was plugged into the medullary canal and pellets were placed in the soft tissues. Some wounds were closed at the time of surgery, negative-pressure therapy was used in wounds difficult to close, and, in case of inadequate skin coverage, wounds were left open to heal via secondary intention.

Our primary outcome measure was the need for higher proximal amputation in the acute treatment phase (six weeks) following surgery. This was chosen as the need for multiple more proximal amputations results in poor outcomes for patients, and the timing was chosen to evaluate the immediate postoperative healing period. Secondary outcomes were healing time, wound infections, debridement or revision of amputation, and duration of follow-up.

## Results

There were 17 procedures performed for 15 patients. Two patients were excluded due to a lack of access to medical notes. Demographic information is shown in Table 1. Peripheral vascular disease was an associated comorbidity in 11 (64.7%) procedures, as was diabetes in 14 (82.4%) procedures. All patients who underwent minor lower limb amputation (transmetatarsal and ray) had diabetes as a comorbidity. There were five below-knee amputations (29.4%), one transmetatarsal (5.9%) and 11 ray amputations (64.7%) (Table 2).

**Table 1:** Demographics of patients undergoing lower limb amputation. Gender and comorbidity are displayed as a percentage of procedures performed.

Demographics	Number	Range/%
Number or patients	15	-
Number of procedures	17	-
Mean age	68	50–89
Males	14	82.4%
Females	3	17.7%
Peripheral vascular disease	11	64.7%
Type 1 diabetes	1	5.9%
Type 2 diabetes	13	76.5%

**Table 2:** Level of lower limb amputation.

Procedures performed	Number	%
Below-knee amputation	5	29.4%
Transmetatarsal amputation	1	5.9%
Ray amputation	11	64.7%

Indications for surgery are shown in Table 3. Some procedures had multiple indications; therefore, the sum of percentages is greater than 100%. Osteomyelitis was the most common indication in 82.4% of procedures, followed by septic arthritis in 17.7%, Charcot joint arthropathy in 17.7%, and 5.9% for necrotising fasciitis.

**Table 3:** Indication for lower limb amputation. As some patients had multiple indications, the sum of percentages is greater than 100%.

Indications for surgery	Number	%
Osteomyelitis	14	82.4%
Septic arthritis	3	17.7%
Charcot joint arthropathy	3	17.7%
Necrotising fasciitis	1	5.9%

The surgical wound was closed in seven (41.2%) procedures and left open or partially closed in 10 (58.8%) procedures. Vancomycin alone was used as the antibiotic in five (29.4%) procedures, gentamicin was used in addition in 11 (64.7%) procedures, and one procedure lacked documentation. Negative-pressure therapy (vacuum-assisted closure) was used in four (23.5%) cases. The mean time to wound healing was 10.5 weeks (open and partially closed wounds, 14.5 weeks; closed wounds, 4.9 weeks). Patients were followed up for a mean of 21.6 weeks.

There were three incidences of wound infection (17.7%), all occurring in minor amputations. No wound infections were noted in major amputations. One patient had a wound infection two weeks following transmetatarsal amputation, which was treated with debridement by podiatry and oral antibiotics. Another patient had two separate wound infections in a particularly difficult healing, partially closed, ray amputation wound, who later went on to undergo vascular intervention. During the inpatient stay, one patient underwent adjacent ray amputation following initial intervention and another subsequent debridement. Other complications included one patient developing a contralateral deep vein thrombosis (5.9%). No adverse effects were noted from the use of Stimulan®. The most commonly isolated bacteria before intervention were *Staphylococcus aureus*, isolated in 35% of cases. Other bacteria included group A beta-haemolytic *Streptococcus*, *Pseudomonas* species, and *Escherichia coli*.

There was no incidence of higher proximal amputations acutely in their recovery period of six weeks following the procedure. One year postoperatively, two (11.8%) procedures had undergone amputation of the adjacent ray. At two years post-surgery, seven (41.2%) procedures had undergone further amputation, with five (29.4%) procedures being an adjacent ray amputation and two (11.8%) having a more proximal amputation. Of these, one patient had a shortening due to a pressure ulcer at six months (who had also previously had an adjacent ray amputated), and one had an above-knee amputation at two years due to vascular disease.

## Discussion

Revision of amputation is a common complication following lower limb amputation, particularly when attempts are made to maintain limb length [11]. It may be due to wound infections, haematoma formation, and wound dehiscence. Many methods have been described to reduce these complications through local antibiotic delivery, which include polymethyl methacrylate beads, collagen sponges, and synthetic bone graft substitutes [10].

Local antibiotic-loaded calcium sulphate has been used previously in amputation. Gentamicin-loaded calcium sulphate with hydroxyapatite (Cerament G) was used in 13 major lower limb amputations. No revisions, surgical site infections, or haematomas were noted within this cohort [10]. Compared to this technique, we used calcium sulphate (Stimulan®) alone both in the medullary canal and in the surrounding soft tissues. The cohort reviewed by Anugraha et al. did not include minor amputations, which could explain the differing rates of wound infection seen in our data (17%). When comparing only the major amputation group in our cohort, the wound infection rate is similar at 0% [10]. Hence, although our study showed no incidence of re-amputation to a more proximal level during acute treatment, the rate of re-amputation is not directly comparable. The increased likelihood of re-amputation in a minor amputation is balanced by the preservation of limb length and functional outcome.

Krause et al. described calcium sulphate beads with tobramycin (OsteoSet-TTM) being placed in the soft tissue of transmetatarsal amputations of 29 patients compared to a control group of 11 patients. They showed a reduced rate of wound breakdown (8.2% vs. 25%) in the antibiotic versus control groups. A longer time to healing was seen in both groups (10.5 vs. 14.5 weeks for beads vs. control) compared to the 4.9 weeks in wounds that were closed in our results. No difference was seen in our overall time to healing (10.5 weeks), which included wounds left open to heal. Similar to our data, no adverse effects were noted by using calcium sulphate beads in the soft tissues. The transtibial revision rates at long-term follow-up were similar between groups (25% beads vs. 27% control). This is higher than the re-amputation rate noted in our results of 0 at a more proximal level in the acute treatment and 5.9% to the transtibial level at two years [7]. This study is again only partially comparable to our cohort due to the exclusion of major and ray amputations. Other studies have shown a range of re-amputation rates in a variety of cohorts: 24% in minor amputations and 7% in major amputations [12]; 1.6% in transmetatarsal and 1.3% in transtibial [13]; and 40% in varying amputations within two years [14].

A prospective, placebo-controlled randomised controlled trial by Monami et al. compared the use of calcium sulphate (Stimulan®) loaded with vancomycin or tobramycin and calcium sulphate without antibiotics placed in the soft tissue of forefoot amputations due to diabetic foot infection (defined as ulcer with osteomyelitis and deep tissue infection) [8]. They built on previous retrospective work showing reduced recurrence of infection, wound breakdown, or progressive amputation compared to matched controls [15]. The prospective study was ceased prematurely when significant superiority was seen in the treatment group. Despite not recruiting to the planned cohort size, five of the ten (50%) patients in the control group (calcium sulphate alone) achieved the primary composite end-point of an infective complication postoperatively compared to none within the treatment group. Of the infective complications in the control group, two went on to have a progressive transtibial amputation [8]. Comparing these outcomes to our cohort is difficult due to the small cohort size from early cessation and differences in cohort demographics. Additionally, the majority of treatment and control groups had toe amputations (65%), a group which was excluded from our study. The acute re-amputation rate in the treatment group is the same as ours at 0%. However, they also saw no wound breakdown or infection, which is lower than the 17.7% wound infection rate seen in our cohort. Comparison of our data with the associated previous work by Ragghianti et al. shows similar infection rates and re-amputation in a 21-patient cohort: 19% reinfection or dehiscence versus our 17.7%; and 0% re-amputation in both [15]. As with the previously mentioned study, the cohort differences in demographics should be considered when comparing to our results.

A wound infection rate of 17.7% was seen in our study in a cohort where 58.8% of surgical wounds were not completely closed. Comparison of this to other studies reporting rates of 34.2% [5] and 69.8% [13], however, is difficult due to the differing cohorts of patients. Venous thromboembolism was seen in 5.9% of our cohort compared to 4% [5] and 1% in other studies [13].

The limitations of our study are a small cohort size without a control and a retrospective design. Heterogeneity of the amputation type and closure method also makes comparison to other literature difficult, but it is pragmatic to the range of uses of this technique. There is also a possibility of potential bias from all patients being under the care of a single consultant surgeon. Absence of prospectively defined parameters for wound healing or infection and incomplete documentation of antibiotic details potentially limit the precision of these results.

Despite the limitations, local antibiotic delivery through Stimulan® is a simple adjunct to amputation, particularly for complex chronic conditions associated with infection. It is intended to seal the medullary canal, which may help reduce blood loss and soakage of the dressing.

## Conclusions

Our method of antibiotic-loaded calcium sulphate placed into the medullary canal and surrounding soft tissues is a technique aiming to reduce the number of wound infections and proximal re-amputation. Our results were derived from a small retrospective cohort of mixed indications. We observed no local or obvious systemic complications following the use of antibiotic-loaded calcium sulphate and no proximal re-amputation within six weeks of surgery. Future studies should aim to assess the outcomes of this technique in a larger number of patients with a prospective design, with the use of controls to add to the evidence for its use. The results aid complex decision-making in amputation, balancing the risk of wound healing complications and maximising function in a challenging group of patients.
